# Lower Avoidant Coping Mediates the Relationship of Emotional Intelligence With Well-Being and Ill-Being

**DOI:** 10.3389/fpsyg.2022.835819

**Published:** 2022-08-09

**Authors:** Carolyn MacCann, Kit S. Double, Indako E. Clarke

**Affiliations:** School of Psychology, The University of Sydney, Darlington, NSW, Australia

**Keywords:** emotional intelligence, depression, anxiety, coping, wellbeing

## Abstract

Emotional intelligence (EI) abilities relate to desirable outcomes such as better well-being, academic performance, and job performance. Previous research shows that coping strategies mediate the effects of ability EI on such outcomes. Across two cross-sectional studies, we show that coping strategies mediate the relationships of ability EI with both well-being (life satisfaction, psychological well-being) and ill-being (depression, anxiety, stress). Study 1 (*N* = 105 first-year university students, 78% female) assessed EI with the Situational Test of Emotion Understanding (STEU) and Situation Test of Emotion Management (STEM). Avoidant coping significantly mediated the relationship of both the STEU and STEM with depression, anxiety, stress, and psychological well-being. EI was associated with lower avoidant coping, higher well-being and lower ill-being. Study 2 (*N* = 115 first-year university students, 67% female) assessed EI with the Mayer–Salovey–Caruso Emotional Intelligence Test (MSCEIT). Avoidant coping mediated the relationship between EI and ill-being, but not the relationship between EI and well-being. These effects were significant for three of the four EI branches—emotion perception, understanding, and management. We discuss possible reasons why avoidant coping may be an active ingredient by which lower EI relates to lower well-being. We also discuss a possible application of our findings—that EI training programs might benefit from including content aimed at reducing avoidant coping.

## Introduction

**Figure 1:**
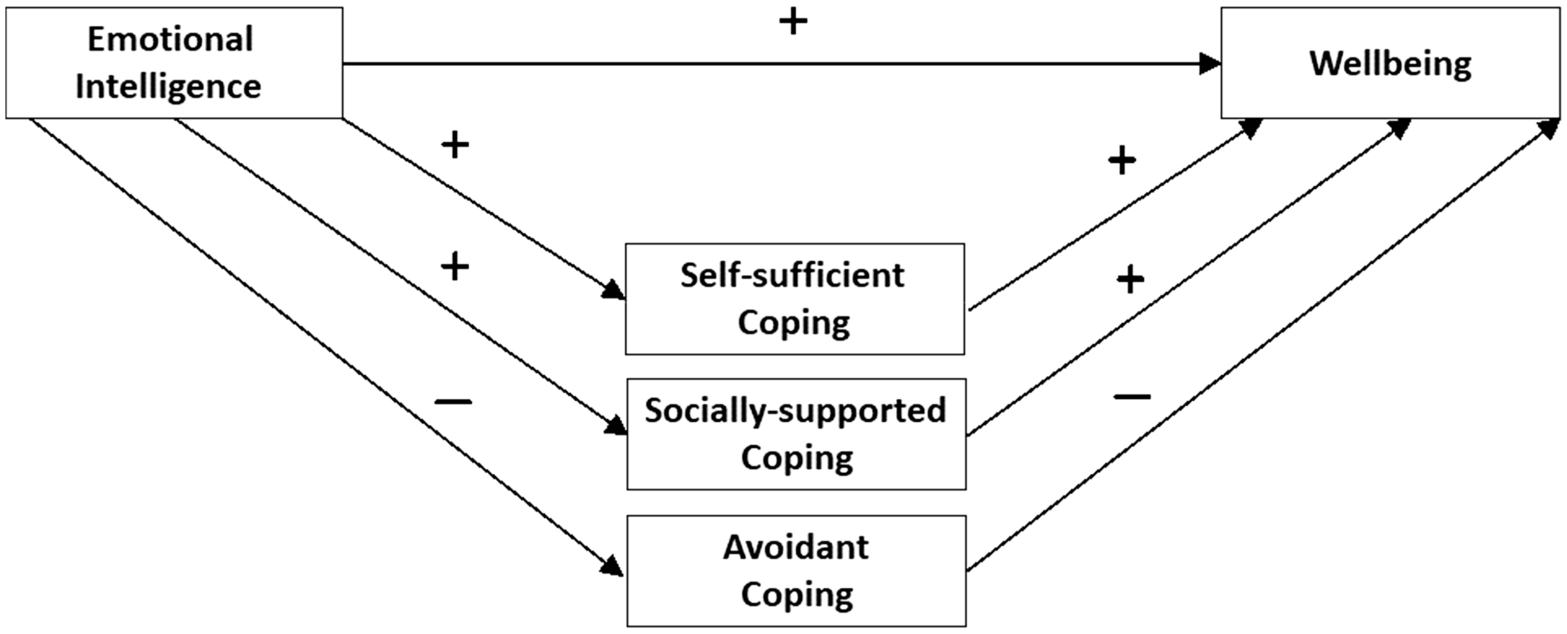
Hypothesized path values when testing a mediation model where coping strategies mediate the effect of EI on well-being outcomes. Signs on the paths indicate the expected directions of the relationships.

Emotional intelligence (EI) consistently shows a positive relationship with a range of valued life outcomes, including job performance, academic performance, mental health, and subjective well-being (Schutte et al., 2007; Joseph and Newman, 2010; Martins et al., 2010; Sánchez-Álvarez et al., 2016; MacCann et al., 2020b). While it is clear that high EI confers benefits to those who possess it, it is not entirely clear what high-EI people do to obtain these benefits. That is, the mechanisms underpinning these relationships are not yet well specified. However, there is evidence that the way that high-EI people cope with stress may account for some of these relationships between EI and valued outcomes. Specifically, coping strategies significantly mediate the relationship between EI and academic performance, marital satisfaction, adolescent depression, well-being, and disruptive behaviors (MacCann et al., 2011; Davis and Humphrey, 2012; Zeidner et al., 2013; Extremera et al., 2020). The current studies add to this body of research by testing whether coping strategies mediate the known relationships of EI abilities with both well-being and ill-being. Across two studies, we examine whether coping strategies mediate the relationship of EI with well-being (psychological well-being, life satisfaction) and with ill-being (anxiety, depression, stress). In the paragraphs below, we define the key concepts we examine (EI, coping, well-being, and ill-being) and provide evidence for our expected associations among EI, coping, and well-being to justify our proposed mediation model (Figure 1).

## Emotional Intelligence

EI was first proposed by Salovey and Mayer (1990) as a set of cognitive skills that allow people to accurately appraise, express, use, and regulate their emotions. Following the publication of Goleman’s (1995) book “Emotional Intelligence,” the rapid growth in the popularity of EI led to multiple differing perspectives on how to conceptualize and measure EI. These multiple perspectives can be classified into two main “types” of EI: a) ability EI (knowledge and information processing of emotions and emotion-related information) and b) trait EI (a set of character traits that underpin social and emotional functioning). Ability EI assessments use objective, maximum-performance tasks (e.g., rating the degree of sadness in a facial expression), whereas trait EI assessments are typical-performance rating scales (e.g., rating one’s agreement with statements like “I can deal well with other people”). Despite sharing the same label, ability EI and trait EI are distinct concepts with minimal empirical and conceptual overlap (e.g., Joseph and Newman, 2010). There is substantially less research on ability EI as compared to trait EI. Meta-analytic summaries report that ability EI research comprises only 22% of studies on EI and coping, 15% of studies on EI and well-being, and 10% of studies on EI and ill-being (Martins et al., 2010; Peña-Sarrionandia et al., 2015; Sánchez-Álvarez et al., 2016). Largely because ability EI is under-studied relative to trait EI, we choose to focus on ability EI in the current research.

For ability EI, there is a single dominant theoretical model—the four-branch hierarchical model of EI (Mayer et al., 2016). The four branches of this model are: (1) *emotion perception*: the ability to accurately perceive the emotions present in facial expressions, tone-of-voice, body-language, or evocative artwork; (2) *emotion facilitation:* the use of emotions to facilitate or aid problem-solving; (3) *emotion understanding*: understanding the way that emotions combine and change over time, and having a good vocabulary of emotional terms; and (4) *emotion management*: successfully regulating the emotions of oneself and others to increase personal well-being or achieve one’s goals. In this paper, we use two different assessments of ability EI to ensure that results generalize across instruments. Study 1 uses the Situational Test of Emotion Understanding (STEU) and the Situational Test of Emotion Management (STEM), which assess the emotion understanding and management of emotions branches of EI (MacCann and Roberts, 2008). Study 2 uses the Mayer–Salovey–Caruso Emotional Intelligence Test (MSCEIT) which assesses all four branches of ability EI (Mayer et al., 2003). Our mediation model (Figure 1) proposes that ability EI is associated with greater well-being and lower ill-being.

## Coping Strategies

Coping refers to the way people respond to stressful situations to reduce their distress. Coping may involve both thoughts (cognitive responses) and actions (behavioral responses) which arise from appraisals of the situation’s personal significance and of one’s capacity to manage it (e.g., a situation may be interpreted as a threat, harm, or challenge to the individual, and this interpretation determines the coping response the individual will make; Folkman, 2013). In this manuscript, we consider habitual ways of coping with daily stressors, assessing individual differences in dispositional coping (i.e., the tendencies to use certain types of coping across multiple situations). We distinguish between the habitual use of three different groups of strategies (Litman, 2006; Litman and Lunsford, 2009). First, *self-sufficient strategies* are intrapersonal strategies that can be implemented independently of other people. These include active coping (taking practical actions to change the situation/stressor, similar to the concept of problem-focused coping) and positive reappraisal (changing the way you think about the situation so that it is less threatening). Second, *socially supported strategies* are interpersonal strategies that involve reaching out to other people for help. These include seeking emotional support (seeking empathy and understanding from others) and instrumental social support (seeking out others’ help to solve a problem). Third, *avoidant strategies* involve disengaging from the situation by ignoring it, withdrawing from it or otherwise avoiding the problem or emotions arising from the situation. These include denial (refusing to acknowledge the reality of a challenging situation) and self-blame (blaming oneself for the situation).

## Well-Being and Ill-Being

While a thorough review of the well-being literature is beyond the scope of the current study (for more comprehensive reviews, see Deci and Ryan, 2008; Dodge et al., 2012), psychologists and economists generally make two key distinctions when describing well-being. First, well-being and ill-being constitute two separate dimensions rather than opposite ends of the same continuum (Headey et al., 1984, 1985). Ill-being includes the experience of anxiety, depression and other negative emotional states representing psychological distress and can be considered the absence or reduced presence of mental health (e.g., Ryff et al., 2006). In contrast, well-being includes the experience of personal fulfilment and positive affective states. Second, there are two distinct aspects of well-being: *eudaimonic well-being* (the psychological well-being tradition; Ryff and Singer, 2008) and *hedonic well-being* (the subjective well-being tradition; Kesebir and Diener, 2008). Hedonic well-being is the experience of pleasure. It can be further divided into *cognitive* elements (such as life satisfaction) and *affective* elements (such as the experience of positive emotions; Diener et al., 1999). Eudaimonic well-being involves self-fulfillment, personal meaning, and feeling that one has reached one’s potential and is often referred to as psychological well-being (Keyes et al., 2002). The two studies in this manuscript both include assessments of hedonic well-being (life satisfaction), eudaimonic well-being (psychological well-being), and ill-being (depression, anxiety, and stress).

## EI and Well-Being

Our mediation model (Figure 1) proposes that EI should relate to higher levels of eudaimonic and hedonic well-being but lower levels of ill-being. We outline the evidence for these relationships below.

### EI and Hedonic Well-Being

Sánchez-Álvarez et al.’s (2016) meta-analysis found that ability EI was significantly related to subjective well-being (*r* = 0.22, *k* = 4), but showed a stronger relationship with cognitive well-being (*r* = 0.25, *k* = 3) than with affective well-being (*r* = 0.14, *k* = 1). Fernández-Berrocal and Extremera’s (2016) narrative review similarly concluded that there was stronger evidence for a relationship of ability EI with cognitive than affective well-being. However, there may be differences across the four branches of EI. Of the four branches, emotion management consistently displays the strongest relationship with cognitive well-being (e.g., Bastian et al., 2005; Law et al., 2008; Ruiz-Aranda et al., 2014). Moreover, emotion management may also show stronger relationships with affective well-being. Meta-analytic findings show that emotion management is the only one of the four branches significantly associated with higher positive affect (MacCann et al., 2020a). In summary, ability EI is significantly associated with hedonic well-being, and effects are stronger for: (a) cognitive versus affective well-being and (b) emotion management versus the other three EI branches.

### EI and Eudaimonic Well-Being

Ability EI is positively associated with eudaimonic well-being (psychological well-being; Brackett and Mayer, 2003; Brackett et al., 2006; Burrus et al., 2012). This association was stronger in a study that used the STEM (*r* = 0.54; Burrus et al., 2012) than in two studies that used the MSCEIT total scores (*r* = 0.28 and 0.19; Brackett and Mayer, 2003; Brackett et al., 2006). There are two possible explanations for this difference: (a) psychological well-being is more strongly related to emotion management than to the other three branches of EI (a construct effect); or (b) psychological well-being is more strongly related to the STEM test than to the MSCEIT test, regardless of the construct (a test effect). By using both the STEM and the MSCEIT in the current research, we can examine whether differences in effect are due to differences among EI branches or differences among tests. We expect that EI will be positively associated with psychological well-being.

### EI and Ill-Being

Two meta-analyses have examined the relationship between EI and mental health (Schutte et al., 2007; Martins et al., 2010). Both considered symptoms of anxiety and depression as indicators of lower mental health and found a small negative relationship between ability EI and these indicators. Schutte et al. (2007) found that the effect was small and nonsignificant (*r* = −0.11, *k* = 4), whereas for Martins et al. a slightly larger and significant effect was observed (*r* = −0.17, *k* = 11). Fernández-Berrocal and Extremera’s (2016) narrative review also found support for a relationship between ability EI and lower depression. In the current studies, we use the 21-item Depression Anxiety Stress Scale (DASS; Lovibond and Lovibond, 1995) as a measure of ill-being. Previous research has found significant correlations between all three DASS scales (depression, anxiety, and stress) and ability EI (MacCann and Roberts, 2008; Doherty et al., 2017; Extremera et al., 2020). Based on the findings discussed above, we expect a small to moderate significant negative association of EI with each of anxiety, depression, and stress.

## EI and Coping

Our mediation model (Figure 1) proposes a positive relationship between EI with both self-sufficient and socially supported coping strategies and a negative relationship between EI with avoidant coping strategies. In the paragraphs below, we outline the evidence for associations of each of these types of coping with ability EI.

This assumption that EI influences the coping strategies people use and the successful implementation of these strategies was assumed in early EI theory (Salovey et al., 1999). In fact, Zeidner et al. (2006) described coping as “emotional intelligence in action” (p. 104), suggesting a link between EI and appraisal-based coping processes, where emotionally intelligent people may cope more effectively due to faster and more accurate processing of emotional material.

Peña-Sarrionandia et al.’s (2015) meta-analysis summarized the associations of ability EI with several coping strategies. They reported significant positive associations of ability EI with self-sufficient and socially supported coping but significant negative associations with avoidant coping. Effect sizes were small for self-sufficient coping strategies (*d* = 0.21 with positive reappraisal and *d* = 0.23 with problem-solving) and moderate for socially supported coping strategies (*d* = 0.50 with social support seeking) and avoidant coping strategies (*d* = −0.41 with denial and *d* = −0.43 with behavioral disengagement). More recent empirical studies report similar findings. Mestre et al. (2017) examined the emotion management branch of ability EI only, finding significant positive associations with active coping strategies such as positive reappraisal and re-focus on planning. Curci et al. (2017) found that ability EI was not significantly associated with a self-sufficient coping strategy (problem-focused coping) but showed significant negative associations with strategies indicative of avoidance coping (emotion-focused and avoidant coping). Both Goldenberg et al. (2006) and MacCann et al. (2011) found that the associations of ability EI with coping strategies were largest for the emotion management branch.

## Coping and Well-Being

While different types of strategies are useful in different situations (e.g., Aldao et al., 2015), evidence suggests that some strategies are more effective than others *on average*. Specifically, self-sufficient and socially supported strategies are generally effective and are linked with positive outcomes, whereas avoidant strategies are generally ineffective and are linked with negative outcomes (Stowell et al., 2001; Abbott, 2003; Moos and Holahan, 2003; Litman, 2006; Litman and Lunsford, 2009). Meta-analytic results suggest that: a) self-sufficient and socially supported coping are associated with greater well-being and lower ill-being, whereas b) avoidant coping strategies are associated with lower well-being and greater ill-being. We summarize these meta-analytic findings below.

For well-being, there are two meta-analyses examining links to coping, one with clinical populations only (Kraiss et al., 2020) and one in general populations (both clinical and non-clinical, Kato, 2015). Kato found that well-being showed a moderate association with self-sufficient coping strategies (active coping and positive re-framing) and socially supported coping but a small negative relationship with well-being. Kraiss et al. found that a self-sufficient coping strategy (positive reappraisal) showed a small to moderate positive association whereas avoidant coping showed a moderate negative association.

For ill-being, meta-analyses by both Aldao et al. (2010) and Kato (2015) examined the relationship of coping strategies to depression and anxiety. Both found a consistent negative relationship between self-sufficient coping strategies (reappraisal, problem-solving, active coping) with all ill-being indices, which varied in size from small to moderate. Kato found little relationship between self-sufficient coping to ill-being. Both found consistent positive relationships of avoidant coping with all ill-being indices that ranged from moderate effect size to moderate to large effect size.

## The Current Research

We propose a conceptual model in which coping strategies mediate the relationship between EI and well-being. The current studies will extend previous studies by examining the link between EI, coping, and well-being by using a broader range of coping strategies, well-being, and ill-being than previously examined. This work will therefore provide a better understanding of how EI impacts a wide range of well-being outcomes, and whether the habitual coping strategies used by high-EI individuals may account for such relationships. The path model shown in Figure 1 indicates the expected directions of the interrelationships among the constructs of interest.

In brief, both the current studies test the same hypotheses, outlined below.

1. **EI will significantly predict coping**, showing positive relationships with self-sufficient coping and socially supported coping (Hypothesis 1a), and a negative relationship with avoidance coping (Hypothesis 1b).
2. **EI will significantly predict well-being and ill-being**, showing a positive relationship with well-being (psychological well-being and life satisfaction; Hypothesis 2a) and a negative relationship with ill-being (depression, anxiety, and stress; Hypothesis 2b).
3. **Coping will significantly predict well-being and ill-being**, with self-sufficient coping predicting greater well-being and lower ill-being (Hypothesis 3a), socially supported coping likewise predicting greater well-being but lower ill-being (Hypothesis 3b), but avoidance-focused coping strategies predicting lower well-being and greater ill-being (Hypothesis 3c).
4. **There will be significant indirect effects of coping on the relationship of EI to both well-being and ill-being**. Specifically, there will be a positive indirect effect through self-sufficient coping (Hypothesis 4a), socially supported coping (Hypothesis 4b), and avoidant coping (Hypothesis 4c).

## Study 1

Study 1 was designed to examine the extent to which the relationship between emotional intelligence and well-being is mediated by coping. We consider both well-being (psychological well-being and life satisfaction) and ill-being (the DASS subscales of depression, anxiety, and stress). We expect that EI will be positively associated with psychological well-being and life satisfaction and negatively associated with psychological distress.

In many of the commonly used instruction sets for coping measures, participants are asked what they would usually do when they are under stress (e.g., Carver et al., 1989) or about a specific stressor that they have experienced in their lives (e.g., McCrae and Costa, 1986). However, different people have different amounts and types of stress in their lives and may therefore be responding in terms of different conceptualizations of what “being under stress” means. For example, the stress of a young child continually absconding from school is a qualitatively different form of stress from job insecurity, which is different again to the stress of a major health or pain condition. In this study, we tried to control for this variation by situating the coping responses within hypothetical situations. Keeping the situation content consistent across all participants effectively controls for variance due to the level of stress people experience in their daily lives. We argue that when the context for coping is held constant, individual differences in dispositional coping can be captured more accurately. This study, therefore, uses 5 scales of the brief COPE as applied to 30 different vignettes (Carver et al., 1989).

This study assesses two of the four branches of EI using two non-proprietary tests of emotional intelligence—the Situational Test of Emotion Understanding (STEU) and Situational Test of Emotion Management (STEM; MacCann and Roberts, 2008) and the measure of coping described below to test whether coping mediates the effect of EI on well-being outcomes. We focus on these two strategic emotional intelligence branches because prior research has demonstrated that these higher branches (and particularly emotion management) show the strongest relationships with coping and well-being (Goldenberg et al., 2006; MacCann et al., 2011, 2020; Ruiz-Aranda et al., 2014).

## Materials and Methods

### Participants

Participants were 105 Australian undergraduate psychology students from The University of Sydney (78.1% female; *M*_age_ = 19.13, SD = 3.56, range = 17 to 51; self-reported ethnicities were 50% White, 46% Asian, 6% other or unspecified). An additional nine participants who took part in the study were excluded from the final analyses due to meeting one or more of the following exclusion criteria: (a) did not complete all of the coping items; (b) endorsed the same response (e.g., “4—Agree”) for all items on a scale (except for the DASS, where endorsing “0” for all items was considered a feasible response); (c) completed the entire test-battery in less than 15 min (a completion time < 15 min was unrealistic and indicative of non-serious responding); or (d) reported speaking English “not at all” or “not well,” such that scores on the EI tests may represent English-language ability rather than EI.

## Measures

### Emotional Intelligence

#### Situational Test of Emotional Management

In this 44-item test, participants are presented with a vignette describing an emotional situation and must rate the effectiveness of each of four strategies for managing that situation (MacCann and Roberts, 2008). For example, “*Evan’s housemate cooked food late at night and left a huge mess in the kitchen that Evan discovered at breakfast. How effective are each of the following actions? (a) Tell his housemate to clean up the mess; (b) Ask his housemate that this not happen again; (c) Clean up the mess himself; (d) Assume that the housemate will clean it later.”* Items were scored based on expert consensus.

#### Situational Test of Emotional Understanding

In this 42-item test, participants must select which of five emotions a protagonist is feeling in a particular situation (MacCann and Roberts, 2008). For example, “*An irritating neighbor of Eve’s moves to another state. Eve is most likely to feel? (a) Regret; (b) Hope; (c) Relief; (d) Sadness; (e) Joy”* (correct answer = relief). Items are scored according to the theoretically correct answer based on Roseman’s appraisal theory (Roseman, 1991).

### Coping

#### Coping (30 Vignettes)

For each of 30 vignettes describing an everyday stressful situation, participants rated how likely they would be to use 10 possible coping responses on a scale from 1 (“Not at all”) to 9 (“Extremely”). These 10 coping responses were taken from five of the brief COPE scales: active coping, denial, use of emotional support, positive reframing, and self-blame, i.e., each strategy had two items (Carver et al., 1989). In keeping with the taxonomy proposed by Litman (2006), the active coping and positive reframing items were averaged to create a “self-sufficient” coping score. The two emotional support coping items were averaged to create a “socially supported” coping score. The denial and self-blame items were averaged to create an “avoidant-oriented” score. An example vignette is “*You have been given a new person to supervise at work. They are not making any progress. Your boss says that you need to use better management skills to help them progress. This person takes up a lot of your time and does not listen to you*.” All 30 vignettes are provided in the open science framework page for this project (https://osf.io/46v9t/). For each vignette, participants rated how they would cope in the situation (6 items). Participants also rated how they would feel in this situation (3 items), but these ratings were not used in the current study.

### Psychological Distress

#### Depression Anxiety Stress Scale (DASS-21)

This 21-item instrument assesses self-reported depression (e.g., “I found it difficult to relax”; 7 items), anxiety (e.g., “I felt I was close to panic”; 7 items) and stress (e.g., “I found it difficult to relax”; 7 items; Lovibond and Lovibond, 1995). Participants responded to each item on a 4-point scale (0 = did not apply to me at all, 3 = applied to me very much, or most of the time) in reference to the past week.

### Well-Being Outcomes

#### Ryff’s Scales of Psychological Well-Being

The 18-item version of this instrument assesses aspects of well-being associated with autonomy, personal growth, relationships, meaning, self-acceptance, and environmental mastery (e.g., “For me, life has been a continuous process of learning, changing, and growth”; Ryff, 1989). Participants responded to each item on a 6-point scale (1 = “Strongly Disagree,” 2 = “Disagree somewhat,” 3 = “Disagree slightly,” 4 = “Agree slightly,” 5 = “Agree somewhat” and 6 = “Strongly Agree”).

#### Satisfaction With Life Scale

This 5-item scale measures global life satisfaction. Items are rated on a 5-point scale from 1 = “Strongly Disagree” to 7 = “Strongly Agree” (e.g., “I am satisfied with my life”; Diener et al., 1985).

### Procedure

Participants made an appointment to attend the in-laboratory study after viewing a study ad on the online experiment participation system (SONA). After reading a participant information form in the laboratory, participants provided written consent to take part in the study. During proctored testing sessions of up to 10 participants, students completed demographic questions and the measures outlined above on university computers. All protocols were programmed in Qualtrics. Students received course credit (2% of their psychology mark that semester) in exchange for participation. This study received ethics approval from The University of Sydney (2014/292).

### Transparency and Openness

Data, scripts, output, and full study protocol are available as electronic supplementary materials at https://osf.io/46v9t/ for both Study 1 and Study 2.

## Analytic Strategy

Hypotheses 1 to 3 were tested with correlation coefficients. Hypothesis 4 was tested as a series of mediation models, performed using the “laavan” R package (Rosseel, 2012). We ran separate mediation models for each of the five DVs (depression, anxiety, stress, psychological well-being and life satisfaction) and ran these separately for emotion understanding and emotion management as predictors. Each model had three mediators (self-sufficient, socially supported and avoidant coping). Standardized beta values are reported in the tables below and path diagrams are available in the supplementary material.^1^ All analyses were performed with R version 3.4 (R Core Team, 2017). We interpret the effect size of correlations and standardized regression coefficients using Cohen’s (1988) guidelines on correlations for what constitutes a small (0.10), medium (0.30) or large (0.50) effect. For the indirect effects, we interpret the effect size in terms of the square root of the *ab* pathway, given that *ab* is a compound effect of *a* and *b* (i.e., small, medium, and large values of *ab* would be 0.01, 0.09, and 0.25, given that small, medium and large values of *a* and *b* would be *r* = 0.10, 0.30, and 0.50).

## Results

### Descriptive Statistics

Descriptive statistics and correlations are presented in Table 1. All scales showed good reliability (Cronbach’s alpha range 0.73 to 0.98).

**Table 1:** Descriptive statistics, reliability, and correlations among EI, coping, ill-being, and well-being outcomes, Study 1 (N = 105).

Variable	*M*	SD	**α**	1	2	3	4	5	6	7	8	9
1. Emotion understanding	0.61	0.13	0.73									
2. Emotion management	0.49	0.08	0.76	0.68^**^								
3. Self-sufficient coping	5.62	1.04	0.96	0.07	0.18							
4. Socially supported coping	4.66	1.39	0.96	−0.07	0.06	0.46^**^						
5. Avoidant coping	3.19	1.21	0.98	−0.40^**^	−0.33^**^	0.19	0.42^**^					
6. Depression	13.05	4.96	0.90	−0.25^*^	−0.24^*^	−0.16	0.03	0.31^**^				
7. Anxiety	11.61	4.09	0.83	−0.48^**^	−0.38^**^	0.06	0.20^*^	0.48^**^	0.54^**^			
8. Stress	14.26	4.47	0.84	−0.28^**^	−0.28^**^	−0.02	0.18	0.36^**^	0.46^**^	0.70^**^		
9. Psychological well-being	4.16	0.60	0.78	0.25^*^	0.29^**^	0.30^**^	−0.04	−0.41^**^	−0.64^**^	−0.39^**^	−0.23^*^	
10. Life satisfaction	4.34	1.34	0.88	0.07	0.05	0.30^**^	0.06	−0.10	−0.55^**^	−0.26^**^	−0.18	0.60^**^

* *p < 0.05;*

** *p < 0.01.*

### Hypothesis Testing

#### Hypothesis 1: EI Predicts Coping

Hypothesis 1 received partial support. Avoidant coping showed significant negative associations with both the STEU (*r* = −0.40) and the STEM (*r* = −0.33). However, there were no significant relationships between EI and either self-sufficient or socially supported coping. These results support Hypothesis 1b but not 1a.

#### Hypothesis 2: EI Predicts Well-Being Outcome

Both the STEU and STEM were significantly negatively correlated with depression, anxiety, and stress (*r* = −0.24 to −0.48) and significantly positively correlated with psychological well-being (*r* = 0.25 and 0.29) but relationships between EI and life satisfaction were not significant. Results were strongest for anxiety (with a large effect size for the STEU and moderate effect size for the STEM), consistent with MacCann and Roberts (2008). These results support Hypothesis 2a and 2b (prediction of DASS subscales and psychological well-being) but not 2c (prediction of life satisfaction).

#### Hypothesis 3: Coping Predicts Well-Being Outcomes

Hypothesis 3 received partial support. Self-sufficient coping significantly predicted both well-being variables but none of the ill-being variables, providing partial support for Hypothesis 3a. Socially supported coping significantly predicted only *greater* anxiety (i.e., the opposite direction to hypotheses), providing no support for Hypothesis 3b. Avoidant coping significantly predicted four of the five outcomes in the expected direction (life satisfaction being the exception), providing some support for Hypothesis 3c. Specifically, avoidant coping was significantly associated with higher scores on the three DASS subscales (with a large effect for anxiety and moderate effects for depression and stress) and with lower psychological well-being (with a moderate to large effect size). There was no significant association with life satisfaction. Self-sufficient coping significantly predicted higher levels of life satisfaction and psychological well-being (with moderate effect size) but was not significantly associated with the DASS scores.

#### Hypothesis 4: Coping Mediates the Effect of EI on Well-Being and Mental Health Outcomes

For each model, Table 2 shows the point estimates for each indirect effect and the total indirect effect of all three coping strategies. Results are described below for emotion understanding and emotion management.

**Table 2:** Indirect effects of EI on ill-being and well-being through coping (fully standardized estimates shown), Study 1, N = 105.

	Depression	Anxiety	Stress	PWB	Life satisfaction
	Estimate (95% CI)	Estimate (95% CI)	Estimate (95% CI)	Estimate (95% CI)	Estimate (95% CI)
**Emotion understanding**					
Direct effect	−0.11	−0.35^**^	−0.16	0.03	−0.03
Total indirect effect	−0.14^*^	−0.13^**^	−0.12^*^	0.22^**^	0.09
Self-sufficient coping	−0.01	0.01	−0.01	0.03	0.02
Socially supported coping	0.01	−0.01	−0.01	0.01	0.01
Avoidant coping	−0.12^*^	−0.13^**^	−0.11^*^	0.18^**^	0.07
**Emotion management**					
Direct effect	−0.10	−0.26^**^	−0.19	0.08	−0.07
Total indirect effect	−0.14^**^	−0.12^*^	−0.09	0.21^**^	0.12^*^
Self-sufficient coping	−0.04	0.01	−0.02	0.07	0.06
Socially supported coping	0.01	0.01	0.01	−0.01	−0.01
Avoidant coping	−0.10^*^	−0.12^*^	−0.09^*^	0.15^**^	0.06

* *p < 0.05;*

** *p < 0.01.*

##### Emotion Understanding

Total indirect effects were significant in four cases (for depression, anxiety, stress and well-being, but not life satisfaction). In all four cases, the indirect effect was significant for avoidant coping but not for self-sufficient coping or socially supported coping. Significant indirect effects ranged in magnitude from moderate (*ab* = 0.11 for stress) to a moderate-to-large effect size for well-being (*ab = 0*.18). That is, mediation occurred through avoidant coping only. These results support Hypothesis 4c (but not 4a or 4b).

##### Emotion Management

Total indirect effects were significant for depression, anxiety, well-being, and life satisfaction (but not stress), and the indirect effect of avoidant coping was significant for four of the five outcome variables (all but life satisfaction). The effect size for the indirect effect of avoidant coping was moderate in all cases. Again, these results support Hypothesis 4c (but not 4a or 4b).

## Study 1 Discussion

The results of Study 1 generally support hypotheses for avoidant coping but not for either of the active forms of coping (self-sufficient coping and socially supported coping). EI predicted lower ill-being and greater psychological well-being (but not greater life satisfaction), and the effect of EI on these outcomes was significantly mediated by avoidant coping. That is to say, the mechanism by which EI affects well-being may be due to less frequent use of ineffective coping strategies rather than more frequent use of effective coping strategies.

## Study 2

The aims of Study 2 were to extend the findings of Study 1 to all four major branches of EI (i.e., to include emotion perception and emotion facilitation, as well the branches of emotion understanding and management used in Study 1). Study 2, therefore, uses the MSCEIT instead of the STEM and STEU (Mayer et al., 2003). The outcome variables are the same as in Study 1, and we test the same hypotheses described above.

## Materials and Methods

### Participants

Participants were 115 undergraduate psychology students from The University of Sydney, participating in exchange for course credit. The sample was 61.7% female with a mean age of 20.10 (*SD* = 3.56), range 17–40. Self-reported ethnicities were 74% White, 21% Asian, 5% more than 1 ethnicity, and 2% other or unspecified. An additional six participants completed some or all study protocols but were excluded using the same exclusion criteria adopted by Study 1 (*n* = 4), or because they had not completed the MSCEIT (*n* = 2).

### Materials

Measures of depression, anxiety, stress, psychological well-being, and life satisfaction were the same as in Study 1. To reduce time demands on participants, we used an abbreviated version of the coping measure in this study, utilizing only 12 vignettes, rather than 30. Four vignettes represented an anxiety-inducing situation, four represented sadness or loss, and four represented irritation/anger.

#### Mayer–Salovey–Caruso Emotional Intelligence Test (MSCEIT)

Emotional intelligence was assessed using a 141-item ability-based assessment of the four branches of emotional intelligence (emotion perception, emotion facilitation, emotion understanding, and managing emotions; Mayer et al., 2003). There were two subtests for each of the four branches. For perception, facilitation, and management sub-tests, participants used a 5-point rating scale (rating the presence of emotion, helpfulness of an emotion, similarly of an emotion to a physical sensation, or effectiveness of response). The emotion understanding tasks are multiple-choice, where participants select one of four options. Consensus scoring was used for all 8 subtests.

## Procedure

As in Study 1, participants made an appointment to attend an in-laboratory proctored testing session after viewing a study ad on the online experiment participation system (SONA). All measures were completed online on university computers, in sessions of up to 10 participants. The coping and well-being assessments were programmed in Qualtrics, and the emotional intelligence assessment was completed *via* the Multi-Health Systems online portal. Students received course credit (2% of their psychology mark that semester) in exchange for participation. This study received ethics approval from The University of Sydney (2013/761).

## Results

All analyses were performed in the same fashion as Study 1. Mediation models were performed separately for each of the four branches of the MSCEIT. Path diagrams are available in the supplementary materials.^2^

### Descriptive Statistics

Descriptive statistics, internal consistency reliability, and correlations for all variables are presented in Table 3. All reliability estimates were reasonable, ranging from 0.68 (for the MSCEIT Facilitation branch) to 0.97 (for socially supported coping). All four MSCEIT branches were positively related (*r* = 0.19 to *r* = 0.56).

**Table 3:** Descriptive statistics, reliability, and correlations among EI, coping, ill-being, and well-being, Study 2 (N = 115).

Variable	M (SD)	α	1	2	3	4	5	6	7	8	9	10	11
1. Perceiving emotions	0.56 (0.09)	0.85											
2. Using emotions	0.47 (0.07)	0.68	0.51^**^										
3. Understanding emotions	0.54 (0.06)	0.74	0.19^*^	0.26^**^									
4. Managing emotions	0.39 (0.07)	0.73	0.31^**^	0.38^**^	0.56^**^								
5. Self-sufficient coping	5.99 (1.15)	0.95	0.03	0.08	0.01	0.21^*^							
6. Socially supported coping	5.28 (1.77)	0.97	0.03	0.16	0.09	0.19^*^	0.49^**^						
7. Avoidant coping	2.99 (1.08)	0.96	−0.25^**^	−0.17	−0.24^**^	−0.28^**^	0.07	0.22^*^					
8. Depression	5.24 (5.00)	0.91	−0.30^**^	−0.24^**^	−0.17	−0.26^**^	−0.17	−0.04	0.34^**^				
9. Anxiety	3.75 (4.08)	0.83	−0.24^*^	−0.27^**^	−0.31^**^	−0.30^**^	−0.12	0.05	0.44^**^	0.68^**^			
10. Stress	7.34 (5.22)	0.87	−0.17	−0.16	−0.20^*^	−0.18^*^	−0.13	0.00	0.38^**^	0.63^**^	0.75^**^		
11. Psychological well-being	13.42 (1.54)	0.69	0.22^*^	0.14	0.13	0.30^**^	0.48^**^	0.15	−0.18^*^	−0.50^**^	−0.23^*^	−0.22^*^	
12. Life satisfaction	4.55 (1.31)	0.87	0.22^*^	0.14	−0.04	0.16	0.29^**^	0.14	−0.14	−0.45^**^	−0.16	−0.16	0.58^**^

* *p < 0.05;*

** *p < 0.01.*

### Hypothesis Testing

#### Hypothesis 1: EI Predicts Coping

Only one of the four branches (emotion management) was significantly related to self-sufficient and socially supported coping, with a small effect size. All EI branches except emotion facilitation were significantly related to lower avoidant coping, with medium effect sizes for the three branches. These results support hypothesis 1b but not 1a, replicating Study 1.

#### Hypothesis 2: EI Predicts Well-Being

EI was significantly related to depression (except understanding emotions), anxiety, and psychological well-being (perceiving and managing emotions) in the expected directions. Two of the four branches (understanding and managing emotions) predicted lower stress, and only one of the four branches (perception) predicted high life satisfaction. Results were moderate for anxiety, small or moderate for depression, small or moderate for psychological well-being, and small for stress and life satisfaction. These results are largely similar to Study 1—the strongest effect occurred for anxiety, with inconsistent relationships to life satisfaction or stress. In general, there was mixed support for Hypotheses 2a and 2b.

#### Hypothesis 3: Coping Predicts Well-Being

As in Study 1, avoidant coping predicted significantly greater depression, anxiety, stress, and significantly lower psychological well-being but not lower life satisfaction. Self-sufficient coping predicted greater psychological well-being and life satisfaction. Socially supported coping was not significantly related to any outcome. In general, results support Hypothesis 3b with much weaker support for 3a.

#### Hypothesis 4: Coping Mediates the EI/Well-Being Relationship

The mediation analyses testing Hypothesis 4 are presented in Table 4. For all four branches of EI: (a) neither self-sufficient coping nor socially supported coping were significant mediators of any outcome, except self-sufficient coping which mediated the EI-psychological wellbeing relationship (b) avoidant coping was a significant mediator in 10 of the 20 analyses (effect sizes were moderate; *ab* = 0.08 to 0.11); and (c) the total indirect effect of EI was significant in 12 of the 20 analyses. These results are largely consistent with Study 1 and largely support Hypothesis 4. Significant results for each branch are given below.

**Table 4:** Indirect effects of EI on ill-being and well-being through coping (fully standardized estimates shown), Study 2, N = 115.

	Depression	Anxiety	Stress	PWB	Life satisfaction
**Perceiving emotions**				
Direct effect	−0.22^*^	−0.13	−0.07	0.17^*^	0.18
Total indirect effect	−0.08^*^	−0.11^*^	−0.10^*^	0.05	0.04
Socially supported coping	0.01	0.01	0.01	0.01	0.01
Self-sufficient coping	−0.01	0.01	0.01	0.01	0.01
Avoidant coping	−0.08^*^	−0.10^*^	−0.09^*^	0.04	0.03
**Using emotions**					
Direct effect	−0.18^*^	−0.21^*^	−0.07	0.17^*^	0.09
Total indirect effect	−0.07	−0.07	−0.10^*^	0.06	0.05
Socially supported coping	0.01	0.01	0.01	−0.01	0.01
Self-sufficient coping	−0.02	−0.01	−0.01	0.04	0.02
Avoidant coping	−0.06	−0.07	−0.09^*^	0.03	0.03
**Understanding emotions**					
Direct effect	−0.10	−0.23^*^	−0.12	0.10	−0.10
Total indirect effect	−0.09^*^	−0.10^*^	−0.10^*^	0.05	0.06
Socially supported coping	0.01	0.01	0.01	−0.01	0.01
Self-sufficient coping	0.01	0.01	0.01	0.01	0.01
Avoidant coping	−0.09^*^	−0.10^*^	−0.10^*^	0.05	0.05
**Managing emotions**					
Direct effect	−0.13	−0.18^*^	−0.05	0.17	0.05
Total indirect effect	−0.13^*^	−0.13^*^	−0.14^*^	0.14^*^	0.11^*^
Socially supported coping	0.01	0.01	0.01	−0.02	0.01
Self-sufficient coping	−0.04	−0.03	−0.03	0.11^*^	0.06
Avoidant coping	−0.09^*^	−0.11^*^	−0.11^*^	0.04	0.04

* *p < 0.05;*

** *p < 0.01.*

##### Emotion Perception

Avoidant coping was a significant mediator for all three DASS scores but not psychological wellbeing or life satisfaction. Total indirect effects were significant for all three DASS scores (but not for psychological well-being or life satisfaction).

##### Emotion Facilitation

Avoidant coping was a significant mediator for stress and no other criterion variable. Total indirect effects were only signficant for stress and no other criterion variable.

##### Emotion Understanding

Avoidant coping was a significant mediator for all DASS scores, but not psychological well-being or life satisfaction. Total indirect effects were significant for the DASS scores.

##### Emotion Management

Avoidant coping was a significant mediator for all three DASS scores, but not life satisfaction or psychological well-being. Indirect effects were significant for all three DASS scores.

## Study 2 Discussion

The results of Study 2 largely replicated Study 1. Avoidant coping, rather than the two forms of active coping, mediated the relationship between EI and well-being outcomes. The results differed somewhat by branch (with emotion understanding showing the strongest effects), and the clearest results were obtained for the DASS, with much less support for the mediation model using life satisfaction and psychological well-being as the outcomes.

## General Discussion

There is considerable evidence that EI abilities predict greater well-being and lower ill-being (e.g., Schutte et al., 2007; Martins et al., 2010; Sánchez-Álvarez et al., 2016; MacCann et al., 2020a). Our results suggest that the EI/well-being relationship can be at least partly accounted for by differences in dispositional coping. Specifically, emotionally intelligent people habitually used less avoidant coping, which was related to lower anxiety, depression and stress, and higher psychological well-being. Across the two studies, there was a consistent indirect effect through avoidant coping that held for all models of ill-being and most models of psychological well-being. There was little empirical support for the effects on life satisfaction or for effects of the other two coping strategies examined (self-sufficient coping and socially supported coping) as mediators.

### Avoidant Coping Is the Critical Ingredient Linking EI to Ill-Being

Across both studies, all branches of EI tended to be related to lower avoidant coping, but were not significantly related to self-sufficient or socially supported coping (except for MSCEIT Management). It is therefore unsurprising that there were consistent significant indirect effects of EI through avoidant coping, but not through self-sufficient or socially supported coping. This is important for understanding the mechanisms linking EI to valued outcomes. There is substantial evidence that the use of avoidant-orientated coping strategies (self-blame, denial) is maladaptive, with a link between the use of these strategies and mood and anxiety disorders well established (e.g., Garnefski et al., 2001; Aldao and Nolen-Hoeksema, 2010). Our results suggest that it is not the adaptive coping strategies high EI people use, but rather the maladaptive coping strategies they do *not* use that relates to their lower ill-being. Specifically, high-EI people *do not* habitually use avoidant coping whereas low-EI people do. This was the major difference and accounted for a significant amount of the relationship between EI with well-being and with ill-being.

There are four possible reasons why avoidant coping may be the critical ingredient linking EI to well-being outcomes. We discuss each of these possible reasons below.

1. **Avoidant coping may be confounded with stressor intensity.** It may well be that avoidant coping (disengaging from the stressor) signifies that the stressor is particularly distressing/unresolvable, such that the intensity of the stressor is the confounding variable linking avoidant coping to lower well-being. For example, individuals who experience highly intense affect tend to use avoidant coping more (Flett et al., 1996) However, we obtained ratings averaged across multiple stressors (12 or 30) that were the same across all individuals so that individual differences in dispositional coping (as we measured it) would not be confounded with individual differences in the severity or type of stressor.
2. **Avoidant coping relates to stress appraisals.** It may be that avoidant coping (disengaging from the stressor) relates to individual differences in stress appraisal. People with low EI may appraise stressors as uniformly lower in coping potential, an appraisal bias thought to underlie hopelessness, helplessness, and potentially anxiety (Scherer, 2009) and so link to avoidance. In both studies, the strongest association of EI with any outcome variable was for emotion understanding with anxiety, which is consistent with this idea (as emotion understanding explicitly involves situational appraisals). Future research could explicitly test whether appraisal biases associated with low EI are the cognitive mechanism linking EI abilities to coping responses.
3. **Other strategies (but not avoidant coping) may require skilled implementation to confer benefit.** It may be that the usefulness of the more active forms of coping (self-sufficient coping and socially supported coping) relies on effective *implementation* of the coping strategy to a greater extent than less active forms of coping (such as avoidant coping). That is, people need to have the *ability* to self-sufficiently cope, not simply the dispositional tendency to do so, for self-sufficient coping to affect well-being. Evidence supports the idea that coping efficacy affects outcomes for active coping but not for avoidant coping (e.g., Frydenberg and Lewis, 2009). In the case of socially supported coping, well-being would also logically be related to the quality and availability of social support (Prati and Pietrantoni, 2009). That is, while it is always helpful to reduce avoidant coping, the helpfulness of self-sufficient and socially supported coping depends on the available resources. Self-sufficient coping is helpful if you have the skills to implement the strategies effectively, and socially supported coping is helpful if you have social support available.
4. **Avoidant coping is known to mediate the effect of personal resources on well-being.** If EI represents a personal resource people can draw from, our results may add to the growing number of findings that avoidant coping mediates the effect of personal resources on well-being. People with greater personal resources are less likely to habitually use avoidant coping, which has positive effects on their well-being. In fact, there is evidence from multiple contexts that avoidant coping mediates the effect of personal resources (or personal burdens) on health and well-being (e.g., Gomez, 1998; Jose and Huntsinger, 2005; Manne et al., 2005; Mausbach et al., 2006; Polman et al., 2010; Boals et al., 2011; Pacella et al., 2011; Cheng et al., 2015; Li et al., 2016; Brooks et al., 2019). In many cases, the indirect effect is found only for avoidant coping and not for other more active forms of coping (Jose and Huntsinger, 2005; Polman et al., 2010; Boals et al., 2011; Brooks et al., 2019). Like other personal resources, an individual’s ability EI may relate to higher well-being through lower use of avoidant coping behaviors (rather than through greater use of particularly active or effective coping strategies).

## Practical Implications of Study Findings

If EI exerts its effects on ill-being through avoidant coping, there are implications for EI training programs. There is clear meta-analytic evidence that EI training programs increase ability EI (Hodzic et al., 2018; Mattingly and Kraiger, 2019), and emerging evidence that they also affect secondary outcomes such as well-being and psychological health (Kotsou et al., 2019). Often, the ultimate goal of EI training is the change to secondary outcomes—decreases in employee stress, student misbehavior, workplace incivility, or a more positive institutional climate. Bluntly put, organizations pay for EI training because they expect that higher EI will have flow-on effects to increased well-being and the identifiable financial benefits associated with this increase (e.g., Mikolajczak and Van Bellegem, 2017). Our results suggest that well-being outcomes could be maximized by including a focus on avoiding sub-optimal responses (i.e., what not to do—avoidant coping) to complement or replace training activities that focus on optimal or ideal responses (i.e., self-sufficient and socially supported coping strategies). Case studies, role plays, and reflection activities might include worst-case scenarios that illustrate the damaging effects of avoidant coping strategies (such as self-blame or denial). While our study did not provide causal evidence of the link between avoidant coping and well-being, Sikkema et al.’s (2013) randomized control trial provides causal evidence that decreasing avoidant coping leads to increases in well-being. In a randomized control trial of a coping intervention, Sikkema et al. (2013) found that training reduced avoidant coping and this reduction completely accounted for the decreases in traumatic stress. The major importance of our research for practitioners is therefore to suggest that emotional intelligence training programs include content on “what not to do,” as lower use of maladaptive coping strategies (such as avoidant coping) may have the strongest effect on well-being and ill-being outcomes.

## Limitations and Future Directions

Both studies used a cross-sectional design with convenience samples of predominantly female psychology undergraduates, which may limit the generalizability of findings. Future research could replicate this model in more diverse samples to test whether results are similar in older samples or to disaggregate by gender to test whether results differ for males and females. Future research could also replicate this model in different contexts (e.g., employment, competitive sport, romantic relationship, or family relationship contexts), and use stronger designs. For example, future research could examine context-specific coping strategies in the EI/well-being relationship as the situation unfolds over time, as coping is a dynamic within-person process (Roesch et al., 2010). Our studies were also limited by the sole reliance on self-ratings of coping and outcome variables, rather than other forms of data (e.g., informant ratings or physiological indices of stress such as cortisol or heart rate variability).

In conclusion, the current studies supported a mediation model whereby EI relates to reduced use of avoidant coping which relates to increased well-being and reduced ill-being. These associations suggest some possible mechanisms by which EI produces greater emotional well-being.

## Data Availability Statement

The datasets presented in this study can be found on the Open Science Framework at: https://osf.io/46v9t/.

## Ethics Statement

The studies involving human participants were reviewed and approved by University of Sydney Human Ethics. The patients/participants provided their written informed consent to participate in this study.

## Author Contributions

CM conceived the study. KD and IC performed the analysis. All authors contributed to the article and approved the submitted version.

## Funding

This research was funded by the Australian Research Council Discovery Project Scheme (DP210103484).

## Conflict of Interest

The authors declare that the research was conducted in the absence of any commercial or financial relationships that could be construed as a potential conflict of interest.

## Publisher’s Note

All claims expressed in this article are solely those of the authors and do not necessarily represent those of their affiliated organizations, or those of the publisher, the editors and the reviewers. Any product that may be evaluated in this article, or claim that may be made by its manufacturer, is not guaranteed or endorsed by the publisher.

## Footnotes

^1^ https://osf.io/vr2zb/

^2^ https://osf.io/vr2zb/
